# Novel *Bacillussubtilis* IND19 cell factory for the simultaneous production of carboxy methyl cellulase and protease using cow dung substrate in solid-substrate fermentation

**DOI:** 10.1186/s13068-016-0481-6

**Published:** 2016-03-22

**Authors:** Ponnuswamy Vijayaraghavan, Arumugaperumal Arun, Naif Abdullah Al-Dhabi, Samuel Gnana Prakash Vincent, Mariadhas Valan Arasu, Ki Choon Choi

**Affiliations:** International Centre for Nanobiotechnology, Centre for Marine Science and Technology, Manonmaniam Sundaranar University, Rajakkamangalam, Kanyakumari, Tamil Nadu 629502 India; Department of Biotechnology, Kalasalingam University, Srivilliputtur, Virudhunagar, Tamilnadu 626126 India; Department of Botany and Microbiology, Addiriyah Chair for Environmental Studies, College of Science, King Saud University, P. O. Box 2455, Riyadh, 11451 Saudi Arabia; Grassland and forage division, National Institute of Animal Science, RDA, Seonghwan-Eup, Cheonan-Si, Chungnam 330-801 Republic of Korea

**Keywords:** Cow dung, Solid-substrate fermentation, Carboxy methyl cellulase, Protease, Multienzymes, Response surface methodology

## Abstract

**Background:**

Hydrolytic enzymes, such as cellulases and proteases, have various applications, including bioethanol production, extraction of fruit and vegetable juice, detergent formulation, and leather processing. Solid-substrate fermentation has been an emerging method to utilize low-cost agricultural residues for the production of these enzymes. Although the production of carboxy methyl cellulase (CMCase) and protease in solid state fermentation (SSF) have been studied extensively, research investigating multienzyme production in a single fermentation process is limited. The production of multienzymes from a single fermentation system could reduce the overall production cost of enzymes. In order to achieve enhanced production of enzymes, the response surface methodology (RSM) was applied.

**Results:**

*Bacillus subtilis* IND19 utilized cow dung substrates for the production of CMCase and protease. A central composite design and a RSM were used to determine the optimal concentrations of peptone, NaH_2_PO_4_, and medium pH. Maximum productions of CMCase and protease were observed at 0.9 % peptone, 0.78 % NaH_2_PO_4_, and medium pH of 8.41, and 1 % peptone, 0.72 % NaH_2_PO_4_, and medium pH of 8.11, respectively. Under the optimized conditions, the experimental yield of CMCase and protease reached 473.01 and 4643 U/g, which were notably close to the predicted response (485.05 and 4710 U/g). These findings corresponded to an overall increase of 2.1- and 2.5-fold in CMCase and protease productions, respectively.

**Conclusions:**

Utilization of cow dung for the production of enzymes is critical to producing multienzymes in a single fermentation step. Cow dung is available in large quantity throughout the year. This report is the first to describe simultaneous production of CMCase and protease using cow dung. This substrate could be directly used as the culture medium without any pretreatment for the production of these enzymes at an industrial scale.

## Background

Cellulases catalyze the hydrolysis of cellulose, and many microorganisms, including fungi, bacteria, and protozoans, to produce cellulase [1]. In recent years, cellulolytic enzymes from *Saccharomyces cerevisiae* [2], *Talaromyces cellulolyticus* [3], and *S. cerevisiae* TJ14 [4] have been identified and characterized for various biotechnological processes. These enzymes have many useful applications in the paper industry, bioethanol generation, extraction of fruit and vegetable juice, textiles, the detergent industry, and animal feed production [5–7]. Proteases are an important group of industrial enzymes and are widely used in the food, chemical, pharmaceutical, and leather processing industries [8]. The global market for these enzymes could reach $4.4 billion by the year 2015, and the maximum sales of industrial enzymes came from the leather and bioethanol market [9]. It was previously reported that the cost of growth medium covered approximately 30–40 % of production cost of industrial enzymes [10]. Hence, simultaneous production of cellulase and protease could help to reduce cost. Research examining novel substrates for the production of cellulase and protease has been a continuous effort.

SSF has been an emerging method to utilize the cost-effective agro-residues to produce cellulases and proteases [11, 12]. In the last two decades, SSF has attracted attention in Western countries due to its advantages in the production of secondary metabolites, enzymes, and novel foods [13]. In SSF, the cheap substrates, such as banana fruit stalk, wheat straw, paddy straw, apple pomace, sugarcane bagasse, oil palm empty fruit bunch, green gram husk, *Imperata cylindrical* grass and potato peel, and pigeon pea, have been utilized for the production of cellulase and protease [14–23]. Although these agro-residues were regarded as the potential substrates in SSF, their availability is largely seasonal. The ideal substrate should be available throughout the year and be cheap. Therefore, cow dung is a possible substrate. Cow dung is rich in cellulose (35.4 %), hemicelluloses (32.6 %), ash (13.3 %), nitrogen (1.4 %), and traces of minerals, such as nitrogen, potassium, and sulphur, and traces of phosphate, iron, cobalt, magnesium, potassium, chloride, and manganese [24].

Most cellulolytic enzymes used in industry are of fungal origin; however, these enzymes lack stability at high temperatures. Because many industrial processes are carried out at high temperatures, there is a need for thermostable enzymes from other sources [25]. Cellulases of bacterial origin have potent activity with crystalline celluloses. These enzymes showed high activity and stability towards alkaline pH and are thermostable in nature compared with the fungal cellulases [26]. Cellulases produced by bacteria are notably high in quantity, whereas the fungal cellulases are mostly inducible in nature [27]. Likewise, a wide range of bacteria are known to produce proteases; a large proportion of the commercially available proteolytic enzymes are derived from the genus *Bacillus* because of their capacity to produce large amounts of alkaline proteases with significant activity and stability at high temperature and pH [8, 28].

The traditional method to evaluate the optimal conditions for enzyme production is based on one-variable-at-a-time approach. However, this approach fails to reflect the interactive effects among the selected factors or variables and it is a time-consuming process and requires multiple experimental runs. Additionally, this method does not guarantee to find accurate optimal conditions. However, statistical methods, such as response surface methodology (RSM), have been greatly used to determine the optimum level of factors in a bioprocess [29, 30]. RSM is a collection of statistical techniques for designing experiments, searching the significant factors, and evaluating optimum conditions, that has been successfully used in the optimization of many bioprocesses [31]. In RSM, 3D plots help to better identify the maximum response and interactions among the tested variables [32]. There have been many studies on RSM-mediated optimization of enzyme production from various microorganisms [33–36].

Well-established enzyme engineering is required for the effective and simultaneous production of multienzymes in a single fermentation [37, 38]. In a multienzyme production system, the supplement of various nutrients are critical, and not all nutrients may enhance the simultaneous production of all enzymes [39]. More than two or three enzymes have been produced in a particular environmental condition by microorganisms, specifically *Bacillus* sp. Multienzyme production is a complex process that is associated with complex patterns of repression and induction resulting from the mixed substrate environment, pH, moisture content, fermentation time, and inoculum concentration in SSF [40]. The interaction among these factors becomes the key aspect for investigation in the multienzyme production in SSF. Several reports are available for *Bacillus* sp. for the production of concomitant enzyme production, including lipase and protease [41], amylase and protease [42], proteases and amylases [43]. However, the reports on simultaneous production of CMCase and proteases from *Bacillus* sp. are limited and perhaps not available. Recently, cow dung was used as the solid substrate for the production of protease [12] and CMCase [36]. To the best of our knowledge, the current study is the first to report simultaneous production of CMCase and protease using cow dung substrate in SSF. Considering the production cost of CMCase and protease, this paper identified the optimum conditions for the production of these enzymes by *Bacillus subtilis* IND19. A statistical approach was employed to identify the significant factors and RSM was used to obtain the optimized conditions for CMCase and protease production in SSF utilizing cow dung substrate.

## Results and discussion

### Screening of *B. subtilis* IND19 for cellulolytic and proteolytic activity

In the present study, seven potential cellulolytic bacterial strains were used, which hydrolysed CMC with the zone range of 3.0–6.0 mm. The bacterial isolates, such as VA1, VA2, VA4, VA5, VA6, and VA7, hydrolysed 5, 3, 4, 5, 3, 3 mm, respectively, on CMC agar plates. The CMCase activity of *B. subtilis* IND19 was higher (6 mm) than the other screened bacterial isolates. Cellulase production of the bacterial strains from the genus *Bacillus* has been reported by various studies [44–46]. The cellulolytic enzyme-producing bacterial isolates, such as VA1, VA2, VA3, VA4, VA5, VA6, and VA7, were evaluated for protease production on skimmed milk agar plates. Among the tested bacterial strain, *B. subtilis* IND19 showed the maximum production of protease on skimmed milk agar plates (12 mm). The other tested isolates showed hydrolytic zone ranging from 3 to 11 mm. Hence, *B. subtilis* IND19 was selected for simultaneous production of CMCase and protease.

### Cow dung is a substrate of choice for simultaneous production of CMCase and protease

In this paper, cow dung was explored as the low-cost substrate for the simultaneous production of CMCase and protease. This low-cost substrate could lower the production cost of enzymes. Because the production of hydrolytic enzymes using different fermentation processes is notably expensive, and the simultaneous production of several industrial enzymes in a single fermentation medium is a great challenge [47]. Cow dung was attempted for enzyme production. The selection of suitable solid waste for any enzyme production in an SSF process mainly depends on the cost and availability of the substrate material [48]. In recent years, many substrates have been reported for the production of CMCase and protease [17, 20, 23, 36, 49]. Considering availability and cost, cow dung is a suitable substrate for the production of cellulase and protease. Reports on SSF of cow dung for the simultaneous production of cellulolytic and proteolytic enzymes using bacteria are limited or perhaps not available. This report could be the first to describe the simultaneous production of CMCase and protease in SSF using cow dung substrate.

### Effect of carbon, nitrogen, and mineral sources on CMCase and protease production

Of the all of carbon sources that were tried, sucrose was the most promising, and the corresponding CMCase activity was 213 ± 34.5 U/g. CMCase productions were 181 ± 15.6, 174 ± 4.6, 148 ± 7.3, and 121 ± 4.8 U/g for maltose, fructose, xylose, and glucose, respectively. Among all carbon sources, sucrose enhanced protease production, and the enzyme activity was 1608 ± 28 U/g. Protease activity levels were 1412 ± 46.4, 1027 ± 46.9, 1092 ± 13.5, and 1358 ± 98 U/g, for maltose, fructose, xylose, and glucose, respectively. Of all nitrogen sources that were tested, peptone was the most promising, and the corresponding CMCase activity was 284 ± 32.7 U/g, and protease activity was 1831 ± 67.4 U/g. CMCase activity levels were 261.5 ± 12.8, 67.5 ± 7.3, 210.5 ± 12.8, and 44 ± 1.5 U/g, for yeast extract, oat meal, beef extract, and ammonium sulphate, respectively. Protease activity levels were 1412 ± 34.8, 913 ± 12.9, 1685 ± 121.5, and 819 ± 38.5 U/g for yeast extract, oat meal, beef extract, and ammonium sulphate. Among the mineral sources tested, sodium dihydrogen phosphate enhanced CMCase (248 ± 18.7 U/g) and protease activity (2113 ± 93 U/g). CMCase activity was 182 ± 7.5, 78 ± 0.6, 147 ± 8.4, 197 ± 18.3, and 136 ± 16.9 for ferrous sulphate, di-sodium hydrogen phosphate, ammonium chloride, sodium nitrate and calcium chloride, respectively. Protease activity was 641 ± 37, 1812 ± 29.5, 1741 ± 33, 1427 ± 20.5, and 1918 ± 33 U/g for ferrous sulphate, di-sodium hydrogen phosphate, ammonium chloride, sodium nitrate and calcium chloride, respectively.

### Screening variables for the production of CMCase and protease by statistical approach

Initial screening of medium components indicated that carbon source (sucrose), nitrogen source (peptone), addition of salt solution (NaH_2_PO_4_), and variation of medium pH induced the CMCase and protease production. A statistical approach (2^5^ full factorial design) was used to identify the most effective variables affecting CMCase and protease production. All experiments were carried out under SSF for 72 h at 37 °C in duplicates. The experimental values of two-level full factorial design for the production of CMCase and protease are given in Table 1. CMCase production varied between 41.5 and 497.4 U/g and protease yield varied from 206.5 to 4778.2 U/g. The variability in the yield of enzyme production in this paper provides space for the optimization of enzyme production. The *F* values of this model for CMCase and protease activities were 49.75 and 75.06 U/g, respectively, which were statistically significant at the 5 % level. In this paper, sucrose, peptone, NaH_2_PO_4_, and the initial pH and moisture content of the culture medium significantly influenced the production of both enzymes (Table 2). These results were in accordance with the observations made with ***C****haetomium* sp. on cellulase production in SSF [50], suggesting that sucrose was the best carbon source for cellulase production. However, cellulose was demonstrated to be the best carbon source for cellulase production from *Bacillus* sp. [51]. Addition of peptone to the cow dung medium positively influenced both CMCase and protease production. Umikalsom et al. [52] recorded peptone as the suitable nitrogen source for the production of cellulase by *Chaetomium globosum* in SSF using delignified oil empty fruit bunch fibre as substrate. Likewise, another report also suggested peptone as the best nitrogen source for the cellulase production from *Marinobacter* sp. MSI032 [53]. In this paper, protease production was enhanced by the supplement of sucrose as the carbon source. This result was in accordance with the observations made with *Yarrowia lipolytica* [54] and *Bacillus* sp. [55]. The *R*^2^ of the model values for the production of CMCase and protease were 0.9970 and 0.9954, and the adjusted *R*^2^ was 0.977 and 0.9821, respectively. The regression equation coefficients of the 2^5^ full factorial models were calculated and the data were well fitted.

**Table 1:** Response of two-level full factorial design for screening of variables for CMCase and protease production

Run	Sucrose	Peptone	NaH_2_PO_4_	pH	Moisture	CMCase activity (U/g)	Protease activity (U/g)
	*A*	*B*	*C*	*D*	*E*		
1	−1	1	1	−1	1	403.8	206.5
2	−1	−1	−1	−1	−1	85.3	1143.8
3	1	1	−1	−1	1	134.21	1547.3
4	1	1	−1	1	−1	252.84	930.6
5	−1	−1	−1	1	−1	130.7	922.9
6	1	1	1	−1	−1	41.5	2030.5
7	1	−1	−1	−1	−1	135.07	2084.8
8	1	1	−1	−1	1	298.53	1875.9
9	1	−1	−1	−1	−1	88.78	1154.7
10	1	−1	−1	−1	1	133.2	916.4
11	1	−1	−1	1	−1	103.9	1143.9
12	−1	1	1	1	1	110.74	2775.8
13	−1	1	−1	−1	−1	129.56	1152.4
14	−1	1	−1	−1	1	228.5	925.3
15	1	1	−1	1	1	399.6	4375.9
16	1	−1	−1	−1	1	123.2	2753.9
17	−1	1	−1	1	1	219.5	1401.6
18	1	1	1	−1	−1	145.12	1170.5
19	−1	1	1	1	−1	441.45	920.5
20	−1	−1	1	−1	1	309.3	1382.7
21	1	−1	1	1	1	150.09	3640.4
22	−1	−1	−1	−1	1	125.8	1106.1
23	1	1	1	1	−1	375.5	915.6
24	1	1	1	1	1	259.6	1210.3
25	−1	−1	1	−1	−1	346.3	1826.6
26	−1	−1	1	1	1	171.5	1154.6
27	1	−1	1	−1	1	108.5	1163.7
28	−1	−1	1	1	−1	66.7	2982.6
29	−1	1	1	−1	−1	90.73	1844.9
30	−1	−1	−1	1	1	145.74	1867.4
31	−1	1	−1	1	−1	497.4	1133.8
32	1	−1	1	1	−1	78.93	4778.2

**Table 2:** Analysis of variance (ANOVA) for the CMCase and protease activity of *B. subtilis* IND19

Source	Sum of squares	df	Mean square	*F* value	*p* value	
Analysis of variance (ANOVA) for the CMCase activity of *B. subtilis* IND19						
Model	4.72E+05	27	1.75E+04	49.75	0.0008	Significant
*A*-Sucrose	1.42E+04	1	1.42E+04	40.46	0.0031	
*B*-Peptone	93049.74	1	93049.74	264.84	<0.0001	
C-NaH_2_PO_4_	3.27E+03	1	3.27E+03	9.31	0.038	
*D*-pH	1.73E+04	1	1.73E+04	49.13	0.0022	
*E*-Moisture	3.24E+03	1	3.24E+03	8.66	0.0423	
*AB*	1.87E+03	1	1.87E+03	5.33	0.0821	
*AC*	5.85E+03	1	5.85E+03	16.64	0.0151	
*AD*	1.18E+04	1	1.18E+04	33.57	0.0044	
*AE*	6.57E+03	1	6.57E+03	18.7	0.0124	
*BC*	8.98E+03	1	8.98E+03	25.56	0.0072	
*BD*	6.36E+04	1	6.36E+04	180.91	0.0002	
*CD*	1.90E+04	1	1.90E+04	54.15	0.0018	
*CE*	4.42E+03	1	4.42E+03	12.58	0.0239	
*DE*	3.30E+04	1	3.30E+04	93.8	0.0006	
*ABC*	3.46E+03	1	3.46E+03	9.86	0.0348	
*ABE*	7.46E+03	1	7.46E+03	21.23	0.01	
*ACD*	1.94E+04	1	1.94E+04	55.17	0.0018	
*ACE*	1.63E+04	1	1.63E+04	46.53	0.0024	
*ADE*	1.91E+04	1	1.91E+04	54.34	0.0018	
*BCD*	2.19E+03	1	2.19E+03	6.23	0.0671	
*BCE*	4.10E+03	1	4.10E+03	11.68	0.0268	
*BDE*	6.52E+04	1	6.52E+04	185.67	0.0002	
*ABCE*	1.36E+04	1	1.36E+04	38.81	0.0034	
*ABDE*	2.15E+04	1	2.15E+04	61.14	0.0014	
*ACDE*	1.35E+03	1	1.35E+03	3.84	0.1216	
*BCDE*	8.84E+03	1	8.84E+03	25.15	0.0074	
*ABCDE*	3.54E+03	1	3.54E+03	10.08	0.0337	
Residual	1.41E+03	4	1.41E+03			
Cor Total	4.73E+05	31	4.73E+05			
ANOVA for the protease activity of *B. subtilis* IND19						
Model	3.36E+07	23	1.46E+06	75.06	<0.0001	Significant
*A*-Sucrose	3.93E+05	1	3.93E+05	20.22	0.002	
*B*-Peptone	2.84E+06	1	2.84E+06	145.89	<0.0001	
C-NaH_2_PO_4_	3.63E+06	1	3.63E+06	186.53	<0.0001	
*D*-pH	2.79E+06	1	2.79E+06	143.25	<0.0001	
*E*-Moisture	2.02E+05	1	2.02E+05	10.37	0.0122	
*AB*	1.65E+06	1	1.65E+06	84.73	<0.0001	
*AC*	2.17E+05	1	2.17E+05	11.14	0.0103	
*AD*	8.21E+05	1	8.21E+05	42.21	0.0002	
*AE*	1.27E+05	1	1.27E+05	6.55	0.0337	
*BC*	9.99E+04	1	9.99E+04	5.14	0.0532	
*BD*	3.81E+06	1	3.81E+06	195.77	<0.0001	
*BE*	2.28E+06	1	2.28E+06	117.25	<0.0001	
*CE*	1.05E+05	1	1.05E+05	5.38	0.0489	
*ABD*	1.11E+06	1	1.11E+06	57.04	<0.0001	
*ABE*	4.54E+06	1	4.54E+06	233.39	<0.0001	
*ACE*	2.27E+05	1	2.27E+05	11.68	0.0091	
*ADE*	2.25E+06	1	2.25E+06	115.41	<0.0001	
*BCD*	1.42E+06	1	1.42E+06	72.82	<0.0001	
*BCE*	5.99E+05	1	5.99E+05	30.8	0.0005	
*ABCD*	6.30E+05	1	6.30E+05	32.38	0.0005	
*ABCE*	1.25E+06	1	1.25E+06	64.02	<0.0001	
*ACDE*	2.42E+06	1	2.42E+06	124.24	<0.0001	
*ABCDE*	1.98E+05	1	1.98E+05	10.17	0.0128	
Residual	1.56E+05	8				
Cor Total	3.37E+07	31				

Final equations in terms of coded factors.

#### CMCase activity

Enzyme activity = +197.86 − 21.08*A* + 53.92*B* + 10.11*C* + 23.22*D* + 9.75*E* + 7.65*AB* − 13.52*AC* + 19.2*AD* + 14.33*AE* − 16.75*BC* + 44.57*BD* − 24.38*CD* − 11.75*CE* − 32.09*DE* + 10.4*ABC* + 15.27*ABE* + 24.61*ACD* − 22.6*ACE* + 24.43*ADE* + 8.27*BCD* − 11.33*BCE* − 45.15*BDE* − 20.64*ABCE* + 25.91*ABDE* + 6.49*ACDE* − 16.62*BCDE* + 10.*52ABCDE.*

#### Protease activity

Enzyme activity = +1700.69 + 110.85*A* + 297.78*B* + 336.7*C* + 295.07*D* + 79.4*E* + 226.93*AB* − 82.29*AC* + 160.17*AD* + 63.11*AE* + 55.88*BC* + 344.94*BD* − 266.95*BE* − 57.21*CE* + 186.2*ABD* + 376.63*ABE* + 84.25*ACE* + 264.85*ADE* + 210.38*BCD* − 136.82*BCE* − 140.29*ABCD* + 197.26*ABCE* + 274.8*ACDE* + 78.62*ABCDE*where *A* is sucrose, *B* is peptone, *C* is NaH_2_PO_4_, *D* is pH, and *E* is moisture.

### Central composite design

Optimizing process parameters was carried out using RSM. The factors—namely, pH, peptone, and NaH_2_PO_4_, which significantly influenced both CMCase and protease production—were selected for further optimization using central composite design **(**CCD) to maximize the CMCase and protease production. Our findings showed that peptone, NaH_2_PO_4_, and pH positively influenced CMCase and protease production. However, an excessive concentration of NaH_2_PO_4_ had a negative effect on protease production. Most cellulases and proteases are inducible enzymes and addition of carbon sources, such as sucrose, mannitol, and maltose, enhanced the production of cellulolytic and proteolytic enzymes [56, 57]. It was previously reported that the production of protease was enhanced by the addition of nitrogen sources, such as tryptone, peptone, yeast extract, skimmed milk, and soybean meal [58]. The observed response in the production of CMCase and protease is shown in Table 3. As shown in Table 4, the *p* value of the model generated was <0.05, suggesting the CMCase and protease activity could be well-described by this model. Analysis of variance (ANOVA) was carried out to establish a response surface quadratic model. The model *F* values of 67.14 and 197.54 implied that both quadratic models for the production of CMCase and protease were significant. The model terms, such as *A*, *B*, *C*, *AB*, *A*^2^, *B*^2^, and *C*^2^, were significant for the production of CMCase; *B*, *C*, *AB*, *AC*, *BC*, *A*^2^, and *B*^2^ were significant for protease production. For CMCase production, the *R*^2^ of the model was 0.9837, indicating that the experimental data agreed well with the model prediction. The model could explain 98.37 % variability observed in the data [59]. In the case of protease production, the *R*^2^ value was 0.9944. The model can explain 99.44 % variability observed in the data. The lack of fit values were 3.17 and 0.9658 for CMCase and protease production, respectively, which were not significant. The signal-to-noise ratios of the models were 24.693 and 46.447, respectively, which indicated an adequate signal for both models. The data obtained from the models were fitted to the following second-order polynomial equation for both enzymes.

**Table 3:** Central composite design of the medium component in coded units for CMCase and protease production

Std	*A*:pH	*B*:Peptone	*C*:NaH_2_PO_4_	Enzyme activity (/g)	
				Cellulase	Protease
1	0	0	0	464	4040
2	−1	−1	−1	270	2080
3	0	0	0	440	4163
4	0	0	1.682	348	3942
5	1.682	0	0	370	1798
6	1	1	1	418	4080
7	1	−1	−1	295	4019
8	0	0	0	442	4530
9	−1	−1	1	180	1728
10	0	0	0	462	4120
11	−1	1	1	362	4686
12	1	1	−1	298	3501
13	1	−1	1	320	398
14	0	1.682	0	434	4000
15	0	0	−1.682	152	4611
16	0	0	0	429	4200
17	−1.682	0	0	127	1608
18	−1	−1	−1	79	3263
19	0	0	0	462	4180
20	0	−1.682	0	252	2109

**Table 4:** ANOVA for the quadratic model for CMCase activity and protease activity of *B. subtilis* IND19

Source	Sum of squares	df	Mean square	*F* value	*p* value	
CMCase activity of *B. subtilis* IND19						
Model	2.75E+05	9	3.05E+04	67.14	<0.0001	Significant
*A*-pH	5.27E+04	1	5.27E+04	116.04	<0.0001	
*B*-Peptone	4.46E+04	1	4.46E+04	98.04	<0.0001	
*C*-NaH_2_PO_4_	3.26E+04	1	3.26E+04	71.81	<0.0001	
*AB*	9.25E+03	1	9.25E+03	20.35	0.0011	
*AC*	2.88E+02	1	2.88E+02	0.63	0.4445	
*BC*	924	1	924	2.03	0.1843	
*A* ^2^	69,800.17	1	69,800.17	153.57	<0.0001	
*B* ^2^	1.89E+04	1	1.89E+04	41.51	<0.0001	
*C* ^2^	6.87E+04	1	6.87E+04	151.24	<0.0001	
Residual	4.55E+03	10	454.51			
Lack of fit	3456.22	5	691.24	3.17		Not significant
Pure error	1.09E+03	5	217.77			
Cor total	2.79E+05	19				
Protease activity of *B. subtilis* IND19						
Model	2.95E+09	9	3.27E+06	197.54	<0.0001	Significant
*A*-pH	2.30E+04	1	2.30E+04	1.39	0.266	
*B*-Peptone	4.82E+0.005	1	4.82E+0.005	291.22	<0.0001	
*C*-NaH_2_PO_4_	7.02E+05	1	7.02E+05	42.35	<0.0001	
*AB*	2.412E+0.05	1	2.412E+0.05	14.55	0.0034	
*AC*	2.12E+06	1	2.12E+06	127.58	<0.0001	
*BC*	8.70E+06	1	8.70E+06	524.67	<0.0001	
*A* ^2^	1.11E+07	1	1.11E+07	670.76	<0.0001	
*B* ^2^	2.31E+06	1	2.31E+06	139.47	<0.0001	
*C* ^2^	1.43E+04	1	1.43E+04	0.86	0.3744	
Residual	1.66E+05	10	16,575.16			
Lack of fit	23,264.15	5	4652.83	0.16	0.9658	Not significant
Pure error	1.43E+05	5	28,497.5			
Cor total	2.96E+07	19				

The final equations in terms of coded factors are as follows.

#### CMCase activity

Enzyme activity = +449.59 + 62.14*A* + 57.12*B* + 48.89*C* − 34*AB* − 6*AC* + 10.75*BC* − 69.59*A*^2^ − 36.18*B*^2^ − 69.06*C*^2^.

#### Protease activity

Enzyme activity = + 4204.52 + 41.04*A* + 594.52*B* − 226.71*C* + 173.63*AB* − 514.12*AC* + 1042.63*BC* − 878.34*A*^2^ − 400.51*B*^2^ + 31.53*C*^2^.

**Fig. 1:**
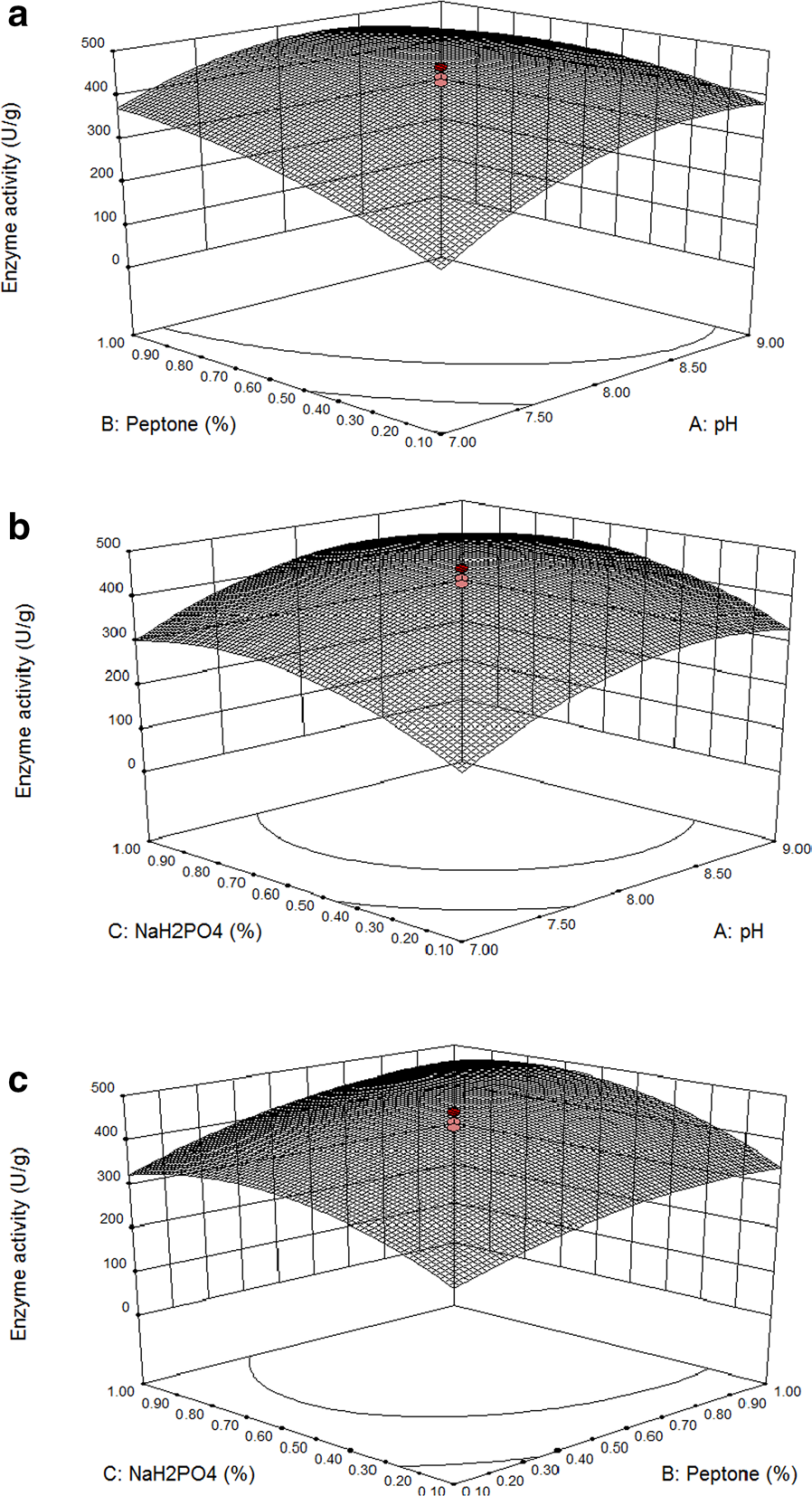
**a** Response surface curve showing the effects of pH and peptone on the CMCase activity of *B. subtilis* IND19 in SSF using cow dung substrate. **b** Response surface curve showing the effects of pH and NaH_2_PO_4_ on the CMCase activity of *B. subtilis* IND19 in SSF using cow dung substrate. **c** Response *surface curve* showing the effects of peptone and NaH_2_PO_4_ on the CMCase activity of *B. subtilis* IND19 in SSF using cow dung substrate

**Fig. 2:**
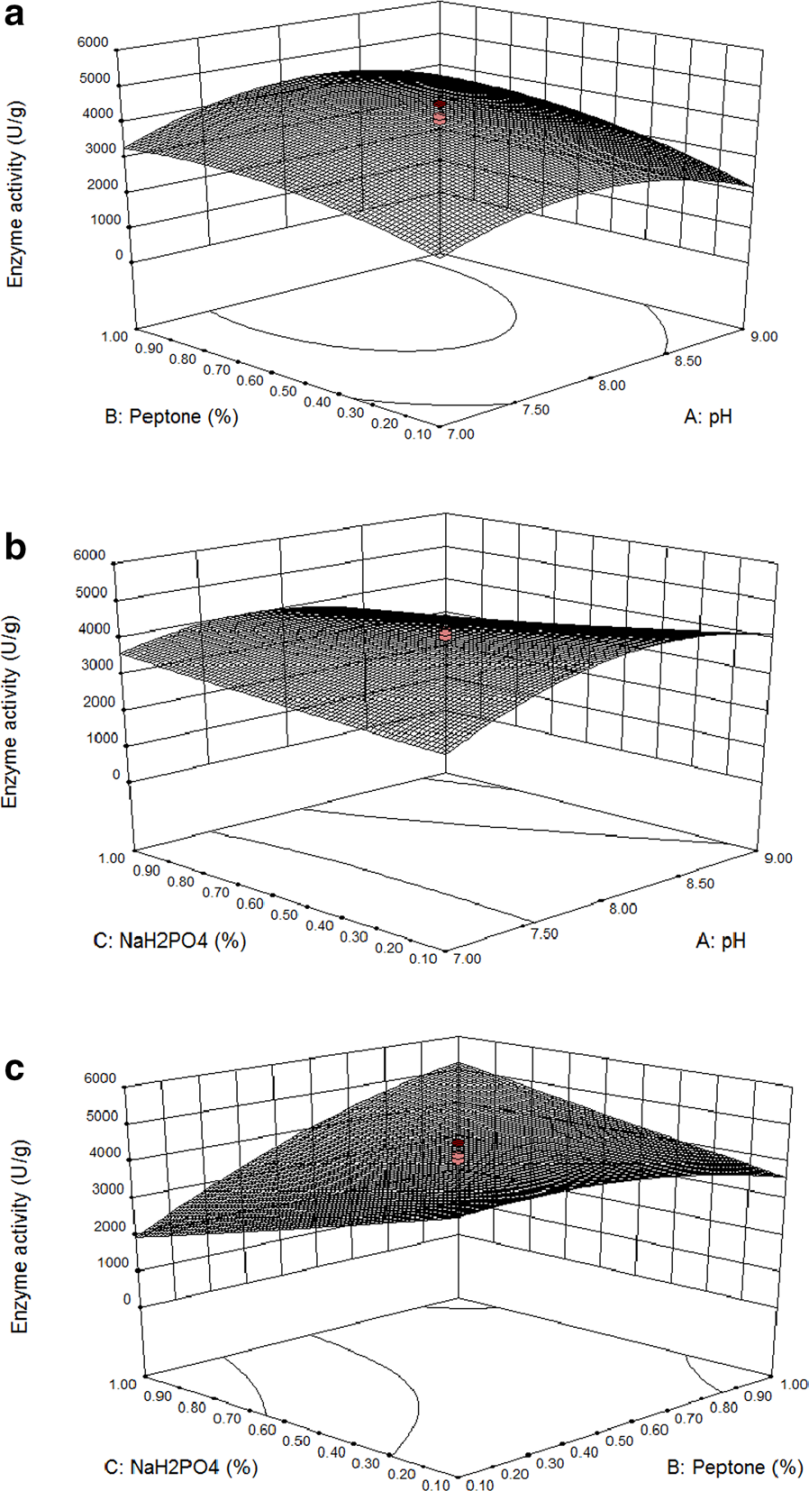
**a** Response surface curve showing the effects of pH and peptone on the protease activity of *B. subtilis* IND19 in SSF using cow dung substrate. **b** Response surface curve showing the effects of pH and NaH_2_PO_4_ on the protease activity of *B. subtilis* IND19 in SSF using cow dung substrate. **c** Response *surface* *curve* showing the effects of peptone and NaH_2_PO_4_ on the protease activity of *B. subtilis* IND19 in SSF using cow dung substrate

The 3D response surface curves in Fig. 1a–c show the interactions among pH, peptone, and NaH_2_PO_4_. These 3D graphs are helpful to identify the interaction between the variables and their levels. The increase in CMCase production was observed in peptone and pH (Fig. 1a), NaH_2_PO_4_ and pH (Fig. 1b), and NaH_2_PO_4_ and peptone (Fig. 1c). However, further increase in all of these three variables beyond the optimized level decreased the production of enzymes. Similarly, the protease production was increased by increasing the concentrations of peptone and NaH_2_PO_4_ (Fig. 2a–c) and was decreased after optimum concentrations of these factors. This was consistent with the fact that CMCase and protease were generally induced in the presence of carbon, nitrogen, minerals, and alteration of pH [18, 23, 36].

RSM has been widely used for the production of enzymes in SSF by various studies [60–63]. It helps to identify the interactive effects of selected parameters and requires the minimum number of experimental runs [64]. In this paper, the maximum CMCase and protease production were observed at 0.9 % peptone, 0.78 % NaH_2_PO_4_, and a substrate pH 8.41, and 1 % peptone, 0.72 % NaH_2_PO_4_, and a substrate pH of 8.11, respectively. Under the optimized conditions, the experimental yield of CMCase and protease reached 473.01 and 4643 U/g, which corresponded to the increase of 2.1-fold and 2.5-fold in CMCase and protease production. This finding could be observed because cow dung is a complex biomass already containing essential nutrients for the growth of microbes [24]. Hence, the addition of nutrient sources merely increased approximately twofold on CMCase and protease production.

### Validation of the experimental model

The response surface model was validated with triplicate experiments under the predicted experimental conditions. The predicted response for CMCase production was 485.05 U/g, which was very close to the experimental value (473.01 U/g), thereby validating this model. The predicted response of the model for the production of protease was 4710 U/g, and the experimental value was 4643 U/g, which validated the model.

### SDS-PAGE analysis of the extracellular protein from *B. subtilis* IND19

**Fig. 3:**
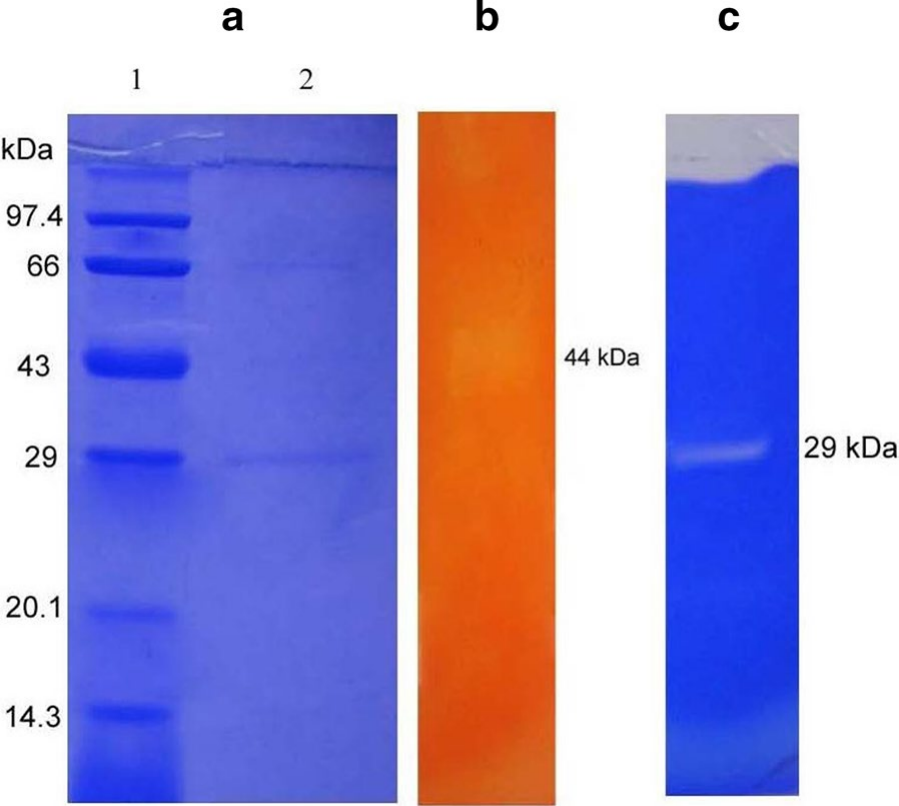
Electrophoresis analysis of the crude enzyme from *B. subtilis* IND19 (*Lane 1*: Protein marker, *Lane 2*: crude protein lysate) (**a**); Zymogram analysis of the crude enzyme for CMCase activity (**b**) and Zymogram analysis of the crude enzyme for protease activity (**c**)

SDS-PAGE analysis revealed the protein pattern from the crude extract of *B. subtilis* IND19 (Fig. 3a). Zymogram analysis of the crude CMCase exhibited a band which corresponds to 44 kDa (Fig. 3b). This result was in accordance with the observations made with other *Bacillus* sp. [65]. The molecular weight of the protease was calculated and was found to be approximately 36.12 kDa (Fig. 3c). The molecular weight of protease was similar to that of the previous reports. Generally, the molecular masses of proteases from various *Bacillus* species range between 17 and 44 kDa [66, 67].

## Conclusions

This study aimed to optimize the simultaneous production of CMCase and protease by *Bacillus subtilis* IND19 with RSM. This report describes the first time that cow dung was applied as the substrate for the simultaneous production of these two enzymes in a single fermentation system. This cheap substrate could be useful for the production of CMCase and protease at industrial scale. RSM-mediated experimental design exhibited an increase of 2.1- and 2.5-fold, respectively, for CMCase and protease compared to non-optimized medium. This paper revealed that RSM is a suitable statistical tool in optimizing enzyme production with minimum experimental runs.

## Methods

### Microorganism

The CMCase- and protease-producing *B*. *subtilis* IND19 was isolated from the soil sample. The isolated *B. subtilis* IND19 was maintained on nutrient agar slants (in g/l) (peptic digest of animal tissue, 5.0; beef extract, 1.5; yeast extract, 1.5; sodium chloride, 5.0; and agar, 15) and stored at 4 °C for further experiments. This organism was subcultured every 30 days.

### Screening of *B. subtilis* IND19 for cellulolytic and proteolytic enzyme

CMCase screening was carried out using carboxy methyl cellulose (CMC) agar medium (in g/l) (beef extract, 5.0; peptic digest of animal tissue, 5.0; yeast extract, 1.5; sodium chloride, 5.0; agar, 15; and CMC, 10). The bacterial growth was visible on these plates after 48 h incubation at 37 °C. To visualize the hydrolysis of CMC agar medium, the plate was stained with Gram’s iodine solution. This formed a bluish black complex with CMC and gave a distinct zone after 5 min [68]. The cellulolytic bacterial isolate, *B. subtilis* IND19 was further grown on skimmed milk agar medium (in g/l) (agar, 15; yeast extract, 5; peptone, 5; KH_2_PO_4_, 1.0; MgSO_4_, 0.2; NaCl, 10; skimmed milk, 10, and pH 10.0). The maximum enzyme-producing bacterial isolate was selected for further studies.

### Molecular identification of the strain

The bacterial isolate was cultured for 18 h in the medium which contained (in g /l): (1) beef extract, 1.5; (2) peptic digest of animal tissue, 5; (3) yeast extract, 1.5; and (4) sodium chloride, 5 (pH 7.0). The genomic DNA of the selected bacterial isolate was purified using a QIAGEN DNA purification kit (Germany) according to the manufacturer’s instructions. The *16S rRNA* gene of *B. subtilis* IND19 was amplified using the upstream primer (P1: 5′-AGAGTTTGATCMTGGCTAG-3′) and the downstream primer (P2: 5′-ACGGGCGG TGTGTRC-3′) (Sigma-Aldrich) [69]. The research gradient Peltier Thermal cycler machine PTC-225 and DNA polymerase (Sigma-Aldrich) were used to amplify the DNA. The following conditions were employed while amplifying DNA: denaturation at 95 °C for 3 min followed by 30 cycles at 95 °C for 1 min, 55 °C for 30 s, and 72 °C for 1 min and 50 s. The amplified 16S rDNA PCR product was sequenced. Further, the identity of the sequences was checked by BLAST through the NCBI server. The 831 bp 16S rDNA sequences of the bacterial isolate were submitted to GenBank and the accession number was assigned (KF688989).

### Inoculum

*B. subtilis* IND19 was grown in the medium which contained (in g/l): (1) beef extract, 5; (2) peptic digest of animal tissue, 5; (3) yeast extract, 1.5; and (4) sodium chloride, 5). The medium was sterilized at 15 lbs for 30 min and cooled. Next, a loopful culture of *B. subtilis* IND19 was inoculated into the 100-ml Erlenmeyer flask. This was incubated on a rotary shaker (175 rpm) at 37 °C for 18 h. This culture was stored at 2–8 °C and was used as the inoculum.

### Substrate

Cow dung was collected from a farm house (Nagercoil, Kanyakumari, Tamil Nadu, India). It was dried for 10 days and powdered. It was stored in an air tight container before further use.

### Production of CMCase and protease under SSF

Cow dung substrate (5 g) was weighed in Erlenmeyer flask (250 ml) and a buffer solution (pH of 8.0, Tris–HCl buffer, 0.1 M) was added to maintain moisture content of the substrate and initial medium pH. Initial moisture content of the medium was maintained as 90 % (v/w). The solid substrate was mixed carefully with buffer and autoclaved at 15 lbs for 30 min and cooled. Then, 10 % inoculum (0.653 OD at 600 nm) was added to the culture medium. The contents were further mixed and incubated for 72 h under 37 °C.

### Enzyme extraction

The fermented medium was stirred with double distilled water (1:10 ratio) and shaken at 175 rpm for 30 min in a rotary shaker. The mixed slurry was then completely filtered using cotton, followed by centrifugation at 10,000 rpm at 4 °C for 10 min. The cell free extract was used as the crude enzyme [49].

### CMCase assay

CMCase activity was assayed using CMC as the substrate. Hundred microliter of crude enzyme was mixed with 100 μL of 1 % (w/v) CMC (pH 7.5) and incubated at 37 °C for 30 min. Next, 1.5 ml of dinitrosalicylate reagent was added, and the mixture was incubated at 100 °C for 10 min. The mixture was cooled, and the absorbance was measured against the reagent blank at 540 nm. One unit of CMCase activity was defined as the amount of enzyme that liberated 1 μmol of reducing sugars per minute under the above conditions [70].

### Protease assay

Casein was used as the substrate for the determination of protease activity. The reaction mixture contained 1.0 ml casein which was prepared in Tris–HCl buffer (0.05 M, pH 8.0) and 0.1 ml of enzyme solution [71]. This mixture was incubated for 30 min at 37 °C and 2.5 ml trichloroacetic acid (0.11 M) was added to terminate the enzyme reaction. It was centrifuged at 10,000* g* for 10 min, and the absorbance of the sample was read against sample blank at 280 nm. One unit of the protease activity was defined as 1 μg of tyrosine liberated min^−1^ under standard assay conditions.

### Screening the optimal carbon, nitrogen, and mineral sources

The effect of carbon sources (1 %, w/w; sucrose, maltose, fructose, xylose, and glucose), nitrogen sources (1 %, w/w; peptone, yeast extract, oat meal, beef extract, and ammonium sulphate), and ionic sources (ferrous sulphate, di-sodium hydrogen phosphate, ammonium chloride, sodium nitrate, calcium chloride, and sodium dihydrogen phosphate) were screened for optimal production of CMCase and protease.

### Elucidation of significant factors affecting CMCase and protease production by statistical approach

Two-level full factorial design (2^5^) was used to identify the significant factors relative to CMCase and protease yield. In this paper, two important physical factors and three nutritional factors were selected. These variables and the selected ranges were based on the results obtained from one-variable-at-a-time approach. The factors selected were sucrose (carbon source), peptone (nitrogen source), NaH_2_PO_4_ (mineral), pH, and moisture (physical factors). Each variable was tested at two levels [high (+) and low (−1)]. In two-level full factorial design (2^5^), a total of 32 experimental runs were generated and the enzyme activities (CMCase and protease) were determined from the crude sample. The variables and their levels are shown in Table 5. The other factors, namely, inoculum size and fermentation period, were kept at optimum level. Two-level full factorial design was based on the χ.

**Table 5:** Variables and their levels for CMCase and protease production using 2^5^ full factorial design

Symbol	Variables	Units	Coded levels	
			−1	1
*A*	Sucrose	%	0.1	1
*B*	Peptone	%	0.1	1
*C*	NaH_2_PO_4_	%	0.01	0.1
*D*	pH	%	6	8
*E*	Moisture	%	90	110

$$Y \, = \, \begin{array}{lllllllll}\alpha _{0} & + & \Sigma \alpha _{i} x_{i} & + & {\text{ }}\Sigma \alpha _{{ij}} x_{i} x_{j} & + & \Sigma \alpha _{{ijk}} x_{i} x_{j} x_{k} & + & \Sigma \alpha _{{ijkl}} x_{i} x_{j} x_{k} x_{l} \\ {} & {} & {i} & {} & {ij} & {} & {ijk} & {} & {ijkl}\end{array}$$

where *α*_*ij*_, *α*_*ijk*_*, α*_*ijkl*_, and *α*_*ijklm*_ are the *ij*th, *ijk*th, *ijkl*th, and *ijklm*th interaction coefficients, respectively, *α*_*i*_ is the *i*th linear coefficient, and *α*_0_ is an intercept.

Assays of CMCase and protease were carried out in triplicates, and the mean value was taken as response (*Y*) (Table 1). ANOVA was used to evaluate the significance of these models, and the *p* value <0.05 indicated that the model terms were significant. Statistical software Design-Expert 9.0.6.2 was used to design the experiments and analyse the results.

### Central composite design and response surface methodology

The CCD was used to identify the optimum concentrations of the factors in order to obtain the maximum CMCase and protease production. The variables selected were analysed at five levels (−*α*, −1, 0, +1, +*α*) (Table 6). According to the Design-Expert 9.0.6.2, for these variables CCD consists of 20 experimental runs including, eight factorial, six axial, and six centre points. Five gram of substrate was taken in 250-ml Erlenmeyer flask, and the required quantities of peptone and NaH_2_PO_4_ were added according to the model. The pH of the medium was maintained according to the model design. The substrate and the supplemented nutrients were mixed carefully, sterilized (121 ± 1 °C for 20 min), and cooled. The Erlenmeyer flasks were inoculated with a 0.5-ml of inoculum (10 %, v/w) and incubated at 37 °C for 72 h. The enzyme was extracted as described previously in the materials and methods. After which, CMCase and protease assays were carried out individually in triplicate. The mean value of the experimental results was considered as response *Y* (Table 3). The fact that values of Prob(>*F*) are smaller than 0.05 would signify that the model terms were significant (Table 4). The experimental results of the CCD were fitted with a following second-order polynomial equation.

**Table 6:** Experimental variables used for optimization of CMCase and protease production in *B. subtilis* IND19

Variables	Symbol	Coded values				
		−α	−1	0	1	+α
pH	*A*	6.32	7	8	9	9.68
Peptone	*B*	−0.21	0.1	0.55	1	1.31
NaH_2_PO_4_	*C*	−0.21	0.1	0.55	1	1.31

$$Y = \alpha _{0} + \alpha _{1}A + \alpha _{2} B + \alpha _{3}C + \alpha _{1} \alpha _{2} AB + \alpha _{1} \alpha _{3} AC + \alpha _{2} \alpha _{3} BC + \alpha _{1} \alpha _{1} A^{2} + \alpha _{2} \alpha _{2} B^{2} + \alpha _{3} \alpha _{3} C^{2}$$

where *Y* is the enzyme activity (U/g); *A* is the coded value of pH; *B* is the coded value of the peptone; *C* is the coded value of NaH_2_PO_4_; *α*_*1*_, *α*_*2*_, and *α*_*3*_ are the linear coefficients; *α*_*1*_*α*_*2*_, *α*_*1*_*α*_*3*_, and *α*_*2*_*α*_*3*_ are the interactive coefficients; and *α*_*1*_*α*_*1*_, *α*_*2*_*α*_*2*_, and *α*_*3*_*α*_*3*_ are the quadratic coefficients.

Response surface graphs were plotted to determine the optimum concentration of factors for the production of CMCase and protease. The fitted polynomial equation was expressed as 3D surface plots to visualize the relation between responses and the experimental levels of each factor used in the design. Validation of the model was performed under the conditions predicted by the model. The predicted response of the model was validated experimentally. Experiments were carried out in triplicates and validated.

### SDS-PAGE and zymogram analysis for CMCase and protease activity

SDS-PAGE was performed using polyacrylamide gel (12 %) [72]. 25 µg crude protein sample was loaded on SDS-PAGE to determine the molecular weight of extracellular protein from *B. subtilis* IND19. A protein marker (97.4–14.3 kDa) was used to determine the molecular weight of proteins. CMCelluose (0.1 %) was co-polymerized to determine CMCase activity and casein (0.1 %) was co-polymerized with SDS-PAGE to determine the protease activity. The sample was not heated before electrophoresis. Zymography analysis was carried out as described previously [73, 74].

## Abbreviations

CMCase: carboxy methyl cellulase
RSM: response surface methodology
SSF: solid state fermentation
NaH_2_PO_4_: sodium dihydrogen phosphate
Na_2_HPO_4_: di-sodium hydrogen phosphate
3D: three dimensional
ANOVA: analysis of variance
CMC: carboxy methyl cellulose
PCR: polymerase chain reaction
OD at 600 nm: optical density at 600 nm
SDS-PAGE: sodium dodecyl sulphate polyacrylamide gel electrophoresis
